# Mid-term results of revision surgery using double-trabecular metal cups alone or combined with impaction bone grafting for complex acetabular defects

**DOI:** 10.1186/s13018-020-01828-x

**Published:** 2020-08-06

**Authors:** Xianghong Zhang, Zhihong Li, Wanchun Wang, Tang Liu, Weiqiu Peng

**Affiliations:** 1 Department of Orthopedics, The Second Xiangya Hospital of Central South University, 139# Middle Renmin Road, Changsha, 410011 Hunan People’s Republic of China; 2 Department of Orthopedics, Liuzhou General Hospital of Guangxi Medical University, Liuzhou, 545000 Guangxi People’s Republic of China

**Keywords:** Revision hip arthroplasty, Acetabular defect, Paprosky III, Double-cup technique, Impacting bone grafting

## Abstract

**Background:**

Revision surgery for complex acetabular defects is still technically challenging. In this study, we discussed and compared the clinical and radiological outcomes of revision surgery between two methods using double-trabecular metal (TM) cups alone or combined with impacting bone grafting (IBG).

**Methods:**

The records of 18 patients (18 hips) who underwent revision surgery using double-trabecular metal (double-TM) cups between 2008 and 2016 were retrospectively reviewed. All the patients were diagnosed with Paprosky III acetabular defects. The acetabular defects were reconstructed by double-TM cups alone or in combination with IBG. We used the modified Harris Hip Score (mHHS), University of California, Los Angeles (UCLA), and Short Form 36 (SF-36) to evaluate the clinical outcomes. Pelvis plain X-ray was used to assess hip center of rotation (COR), abduction angle and anteversion angle of acetabular cup, and incorporation of the bone graft to host bone.

**Results:**

The median follow-up time was 61.0 (IQR 56.0 to 65.8) months. No patients underwent re-revision for loosening or any other reasons. Complications included 3 patients (16.7%) with early dislocation and 3 patients (16.7%) with delayed wound healing. The average mHHS and UCLA preoperatively were 44.1 ± 4.0 (range 35 to 50) and 2.6 ± 0.7 (range 2 to 4), respectively and at the last follow-up were 73.7 ± 4.2 (range 68 to 85) and 7.3 ± 0.5 (range 7 to 8), respectively. The mean SF-36 scores at the last follow-up were improved significantly than preoperative scores, especially in bodily pain category (*P* < 0.05). The average limb-length discrepancy (LLD) decreased significantly from 24.2 ± 2.6 (range 20 to 32) mm preoperatively to 5.8 ± 1.8 (range 3 to 9) mm at the last follow-up, respectively. However, there was no significant difference between two methods at the last follow-up in terms of mHHS, UCLA, SF-36, LLD, and hip COR (*P* > 0.05). Radiographic evaluation demonstrated bone graft incorporation in all hips in the follow-up.

**Conclusions:**

Defect reconstruction using double-TM cups alone or combined with IBG are practical and reliable treatment options for Paprosky III acetabular defects without pelvic discontinuity. Nevertheless, high postoperative complication rate, especially in terms of dislocation, remains a challenge.

## Background

Because of good to excellent prognosis, primary total hip arthroplasty (THA) is an effective treatment for advanced hip diseases [1]. The failure rate requiring reoperation after primary THA is up to 12% at 10-year follow-up [2, 3]. With the younger tendency and the rising life expectancy of patients undergoing THA, the amount of revision surgery following THA is expected to increase in the near future [4]. The restoration of the native hip center of rotation (COR) plays an important role in primary THA and revision THA [5, 6]. Various types of treatments and implants for the reconstruction of acetabular bone defects have been developed recently [2, 7]. On account of advantages in biomechanics and biocompatibility, trabecular metal (TM) augments and TM cup were increasingly used in revision THA [8].

Bone grafting was required to reconstruct the complex periacetabular bone defects [9]. The technique of impacting bone grafting (IBG) would take the place of the bulk grafts gradually because of low osseointegration potential of bulk grafts [10]. Furthermore, it has been reported that the revision THA using structural bone graft without reinforcement devices can lead to a poor result [11]. Regardless of the method used, the proper anchoring would be hindered in severe acetabular bone defects [10]. Some researchers suggested that the technique of using custom-made implants in combination with TM component was a reliable option to deal with complex acetabular defects [7, 12]. However, there is no gold standard for the treatment of complex periacetabular bone defects, and acetabular revision for severe bone defects is still a challenging surgery [11, 13]. In order to increase the function results after reconstruction of acetabular bone defect, new treatment options of using IBG and other revision devices were recommended [14].

In the present study, we extended the use of double-trabecular metal (double-TM) cups alone or combined with IBG to revision surgery in patients with Paprosky III acetabular defect without pelvic discontinuity. The purpose of the current study was to compare and analyze the clinical and radiological outcomes of these two methods for revision surgery in complex acetabular defects. We hypothesized that using double-TM cups alone or combined with IBG were dependable techniques to manage Paprosky III acetabular defects without pelvic discontinuity.

## Patients and methods

### Patients

This retrospective study was approved by the Ethics Committee on Human Research of the Second Xiangya Hospital of Central South University, and written informed consent was obtained from the patients or their legal guardians. A retrospective study including patients with hip revision was performed from January 1, 2008, to December 31, 2016. Aseptic loosening was diagnosed by the uniform standard of clinic and radiologic [7]. Chronic instability was defined as Sayac et al. reported [4]. Musculoskeletal Infection Society (MSIS) was used to diagnose periprosthetic joint infection (PJI) [15], and PJI patients were all treated with two-stage revision. Based on the Paprosky classification [16], acetabular bone defects were identified and categorized according to radiographic and intra-operative findings by senior orthopedic surgeons. The inclusion criteria are as follows: (1) revision surgery with Paprosky III acetabular bone defects and (2) using double-TM cups alone or combined with IBG in acetabulum reconstruction. The exclusion criteria are as follows: (1) patients with hip bone tumors, (2) follow-up period less than 3 years, and (3) patients with incomplete medical records.

**Fig. 1:**
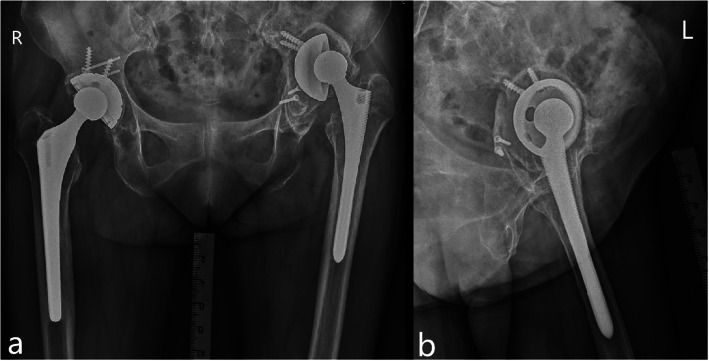
Preoperative anteroposterior (**a**) and lateral (**b**) radiograph show a 74-year-old female presented with Paprosky IIIa acetabular defects after primary THA

There were 386 patients (403 hips) who have undergone hip revision surgery in our department, among which 81 patients (85 hips), 51 patients (53 hips), and 254 patients (265 hips) were acetabular revision, femoral revision, and revision THA, respectively. Our present retrospective study involved 21 patients (21 hips) who underwent Paprosky III acetabular defects and revision surgery using double-TM cups alone or combined with IBG, among which 18 patients (18 hips) were available for complete follow-up data, and 3 patients (3 hips) were lost to follow-up because of natural death or out of touch. Ten (55.6%) female and 8 (44.4%) male patients were included with a medians body mass index (BMI) of 25.9 (IQR 24.3 to 26.0) kg/m^2^, and a median age of 67.5 (IQR 65.3 to 69.0) years at the time of revision surgery, and a median follow-up time of 61.0 (IQR 56.0 to 65.8) months. Indication for acetabular revision of patients was aseptic loosening (100%). The included patients had undergone between 1 and 3 previous surgeries before current revision procedure. Eight (44.4%) patients underwent revision surgery with the use of double-TM cups combined with IBG. The clinical and radiographic data (Fig. 1) about included patients were collected under the same criteria. Demographic characteristics of our included patients were showed in Table 1.

**Table 1:** Patient demographics

Variables	Total cases	Double-TM cups alone	Double-TM cups combined with IBG	***p*** value
No. of patients (%)	18 (100.0)	10 (55.6)	8 (44.4)	-
Sex (No. %)				0.606
Male	8 (44.4)	5 (50.0)	3 (37.5)	
Female	10 (55.6)	5 (50.0)	5 (62.5)	
Age (median, IQR) (year)	67.5 (65.3–69.0)	67.0 (64.3–68.3)	68.5 (66–73)	0.229
BMI (median, IQR) (kg/m^2^)	25.9 (24.3–26.0)	25.3 (24.3–25.9)	26.0 (25.4–26.4)	0.155
Previous surgeries (median, IQR)	1.0 (1.0–2.0)	1.0 (1.0–2.0)	1.0 (1.0–2.0)	0.796
Type of surgery (No. %)				0.744
Acetabular revision	6 (33.3)	3 (30.0)	3 (37.5)	
Revision THA	12(66.7)	7 (70.0)	5 (62.5)	
Paprosky Type (No. %)				0.401
IIIa	11 (61.1)	7 (70.0)	4 (50.0)	
IIIb	7 (38.9)	3 (30.0)	4 (50.0)	
Follow-up (median, IQR) (months)	61.0 (56.0–65.8)	59.0 (56.0–64.5)	63.0 (60.3–67.8)	0.286

*TM* trabecular metal, *IBG* impacting bone grafting, *IQR* interquartile ranges, *BMI* body mass index, *THA* total hip arthroplasty

### Surgical technique

**Fig. 2:**
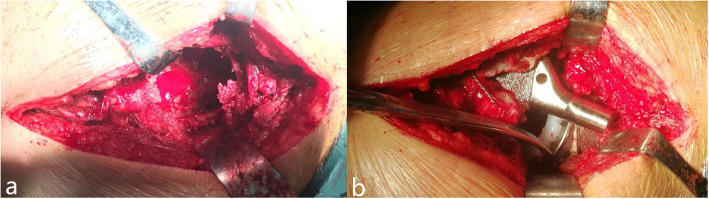
Acetabular bone defects were quantified and categorized during operation (**a**). Creating one monolithic construction after using bone cement between two acetabular cups (**b**)

**Fig. 3:**
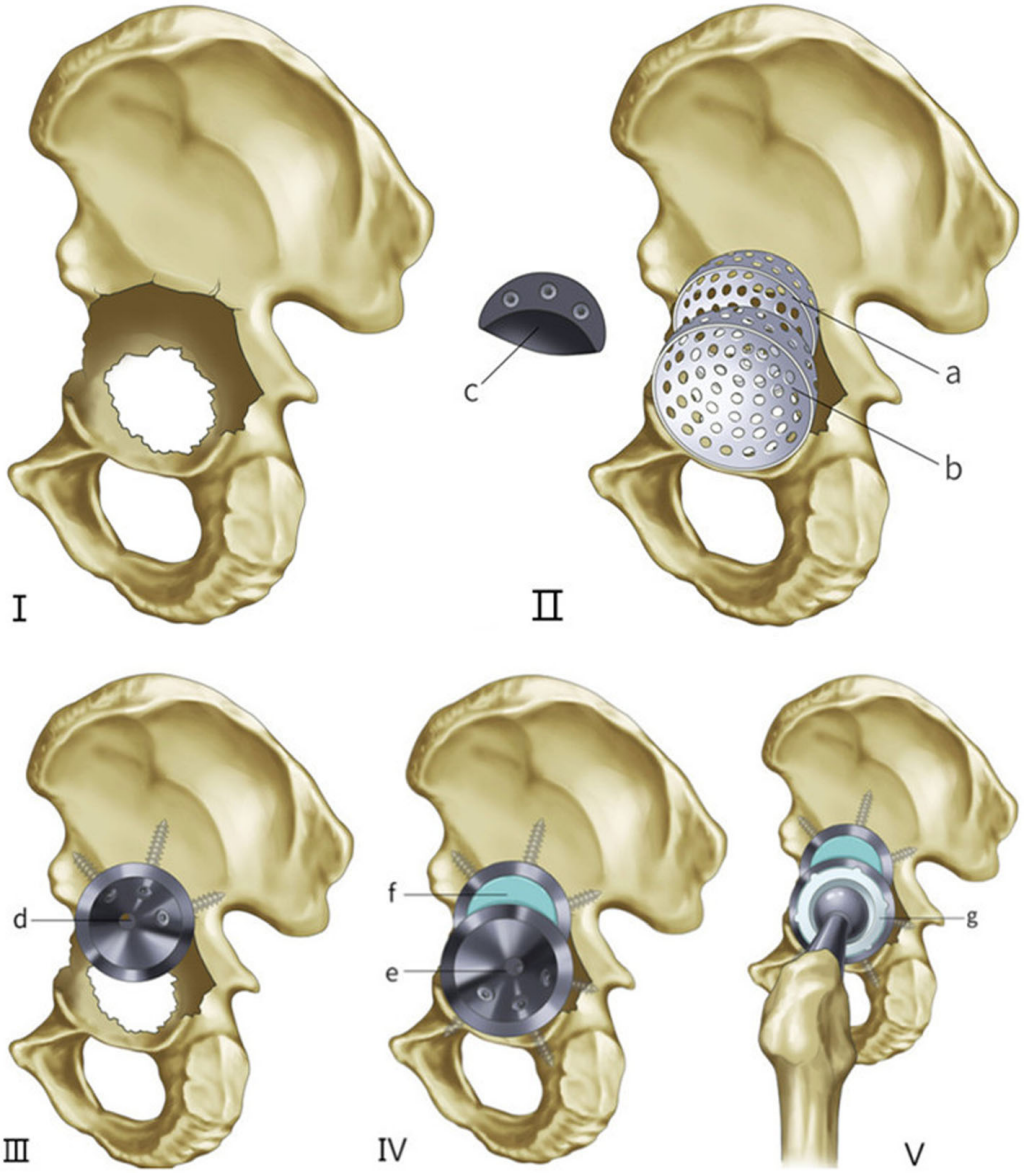
Surgical technique. **I** The complex acetabular defects. **II** Achieving the appropriate size (a, b, c) and position of prosthesis by trials. **III** Placing the 2nd acetabular cup (d) in the posterosuperior defects. **IV** Placing the 1st acetabular cup (e) and using bone cement (f) to create one monolithic construct. **V** inserting the polyethylene liner (g) into the 1st acetabular cup

Revision surgeries were all carried out by posterolateral approach of previous surgery after laying patients in lateral decubitus position. All the patients were operated by or under the direct supervision of the senior authors (WCW and ZHL). We removed the interface membrane and cleaned the acetabulum after explanting the existing cup. The acetabular defects were quantified and categorized by Paprosky grading [16] and recorded at the time of surgery (Fig. 2a, Fig. 3I). Our present study included 11 (61.1%) patients with Paprosky IIIa defects and 7 (38.9%) cases with Paprosky IIIb defects without pelvic discontinuity. In order to achieve appropriate size and position of the prosthesis, an initial trial of the preoperatively proposed double-TM cups construct could be made by placement of a posterosuperior trial shell combined with another optimal trial shell based on the remaining bone defects before reaming (Fig. 3II). The acetabulum was prepared using hemispherical reamers and burrs to reveal bleeding bone. Attention should be paid to avoiding the aggravation of acetabular defects and to preserving the residual structure of the acetabulum.

In some patients with severe bone defects, allograft bone (OsteoRad, Shanxi, China) was used to re-create the acetabulum by impaction technique [17–19]. The allograft chips of an adequate size were routine prepared by hand. All areas of the cavity and cysts were soundly packed with the use of suitable diameter allograft chips and bone graft was then introduced into the socket [18]. Multiple impactions were made ensuring that the final graft surfaces were tightly packed and should feel like cortical bone [18, 19]. Once a tight impaction had been achieved, a trial of the proposed double-TM cups construct could be made once again with the help of intra-operative radiographs.

When achieving a desired position and initial stability, we implanted the revision TM cup as the 2nd acetabular cup (Zimmer, Warsaw, IN) with or without augment in the posterosuperior defects as a support base for stabilizing the 1st acetabular cup (Fig. 3III). Adjunctive screws (Zimmer, Warsaw, IN) were used to increase the initial stability of the 2nd acetabular cup. Before impacting the 1st acetabular cup, the position was tried again by hemispherical reamer. We evaluated the position of acetabular cup through intra-operative plain radiographs. The 1st acetabular cup was desired to be oriented at 40° ± 10° abduction angle and 15° ± 10° anteversion angle [20]. The 1st revision TM cup was then inserted into the pre-rehearsed position of abduction and anteversion by press-fit technique (Fig. 3IV). Finally, we routinely used screws to fix the acetabular cup to the acetabulum. After the acetabular components were fixed, the clindamycin and gentamicin polymethyl methacrylate bone cement (Copal G+C, Berlin, Germany) were used to create a monolithic construct (Fig. 2b, Fig. 3IV). We cleaned the TM cup and inserted the polyethylene liner into the 1st acetabular cup (Fig. 3V). Femoral revision was then carried out as required in 12 of the 18 patients. Adjunctive screw fixation (median 5; IQR 4.25 to 5.25) was used in all patients. Metal-polyethylene coupling was use in 7 hips, while in 11 hips ceramic-polyethylene coupling was used. Femoral head diameter was 32 mm in all patients.

Postoperatively, all patients were given oral celecoxib (200 mg/day) prophylactically for anti-inflammation. At the time of surgery in every revision, samples were sent for microbiologic culture and histologic examination to identify and exclude the infection. For aseptic revision surgery, a second-generation cephalosporin was transfused at least 30 min before skin incision and continued less than 48 h after operation. Targeted prophylactic antibiotic of PJI patients was transfused at least 30 min before skin incision and continued 7–10 days until the negative results of microbiologic culture [21]. In order to avoid deep vein thrombosis and pulmonary embolism, anti-embolism exercises were routinely used immediately, and chemical anti-embolism drugs were administered when indicated according to serum blood coagulation profiles. Early postoperative mobilization was allowed on the first or second day after operation, and part weight-bearing was permitted at early stage of 6–8 weeks after surgery. Full weight-bearing was permitted gradually after the clinical and radiological results were reviewed two months after revision surgery.

### Clinical assessment

All patients were evaluated clinically before operation, and patients were evaluated clinically every month within 3 months after the revision surgery, and after 6 months, 1 year, and then annually during the follow-up period. Trendelenburg sign was applied to evaluated abduction strength. We applied the modified Harris Hip Score (mHHS) for the clinical and functional evaluation, and the mHHS was considered the primary outcome parameter and classified as previous described [22]. In addition, measurements like University of California, Los Angeles (UCLA) activity score [23] and Short Form 36 (SF-36) [10] were recorded. The distance from the anterior superior iliac spine to the medial maleolus was measured as the leg length, and the difference between both lower extremities was calculated and defined as limb-length discrepancy (LLD) [22, 23].

### Radiographic assessment

**Fig. 4:**
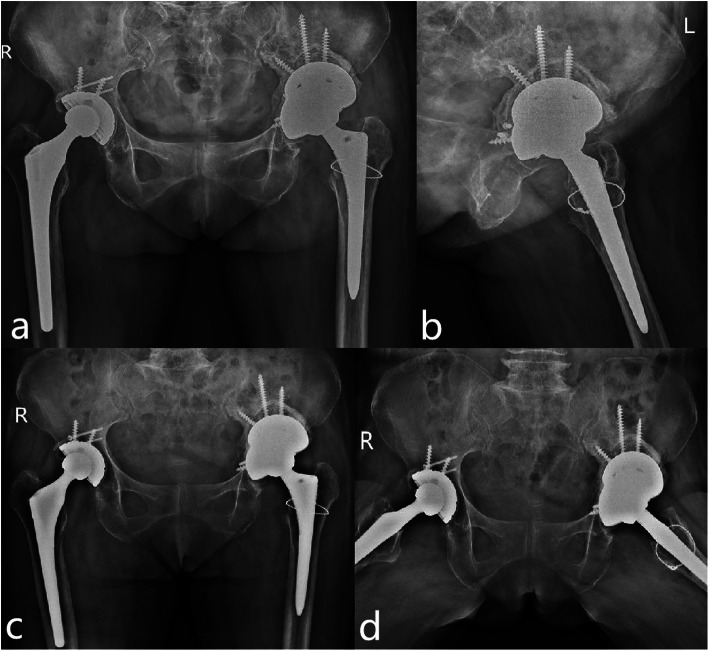
Radiographs (**a**, **b**) show double-TM cups buttress bring the hip COR to an anatomic position. Postoperative radiographs (**c**, **d**) show no prosthesis loosen or dislocation at the 1 year follow-up after revision surgery

All patients were assessed radiologically every month within three months after the revision surgery, and after 6 months, 1 year, and then annually during the follow-up period (Fig. 4). Standard anteroposterior radiographs of the pelvis and later radiographs of the affected hip were performed at each follow-up visit. Radiolucent lines surrounding the implanted components were assessed in accordance with the previous method [22], and loosening of acetabular prosthesis was defined as Sporer and Paprosky reported [24]. The criteria about osseointegration [13] and the Oswestry classification [4] were separately applied to evaluate ingrowth of uncemented component and the integration of allograft bone, and any heterotopic ossifications were noted and evaluated based on the system of Brooker et al. [25]. Two independent authors retrospectively reviewed and evaluated radiological outcomes blindly to minimize bias and the inconsistent evaluation was resolved through discussion and consensus with additional two senior surgeons. The vertical and horizontal position of COR were defined as the distance from the COR to the inter-teardrop line and the distance from the COR to the floor of the acetabular teardrop, respectively [26]. We measured and calculated the acetabular cup abduction angle and anteversion angle on pelvic plain radiographs according to Lewinnek et al. [20].

### Statistical analysis

All the data were analyzed by SPSS 19.0 software (SPSS Inc., Chicago, IL, USA). We used the Kolmogorov-Smirnov test to determine whether the data followed a normal distribution. The non-normal distribution data were described as medians (IQR, Q1–Q3), while normal distribution data were described as mean ± standard deviation. The enumeration data were described as count and rate or percent. The Mann-Whitney *U* test was used to analyze difference between preoperative and the last follow-up values of mHHS, UCLA, SF-36, LLD, and COR. We considered it statistically significant when the *p* value was less than 0.05 for all tests.

## Results

A total of 18 cases out in 21 patients in this group were followed up at last, with a follow-up rate of 85.7%. The median operative time and blood loss volume during the operation were 210 (IQR 201 to 240) min and 950 (IQR 900 to 1013) ml, respectively. There was no significant difference between these two methods in terms of age (*p* = 0.229), sex (*p* = 0.606), BMI (*p* = 0.155), previous surgeries (*p* = 0.796), and follow-up period (*p* = 0.286) (Table 1). No patients experienced infection and nerve palsy after the revision surgery. Complications of dislocation were treated with closed reduction and bracing, and delayed wound healing was treated with prolonged wound dressing. The dislocation rate of acetabular revision (33.3%) was higher than revision THA (8.3%). Trendelenburg-positive was showed in 2 (11.1%) hips at the last follow-up. No patients underwent re-revision surgery for any reasons at the last follow-up (Table 2).

**Table 2:** Review and compare the literature of Paprosky III acetabular defects revision using double-cup technique

Authors	Number of patients	Male/female	Age (years)	Paprosky classification	Follow-up	mHHS of the last follow-up	Survivorship of the last follow-up	Complications
**Webb et al. **[7]	20	9/11	67	Type 3a (55%); type 3b (45%)	28.8 months	68.7	No failure	Complication incidence (40%), dislocation (30%), delayed wound healing (15%)
**Loppini et al. **[12]	16	5/11	68	Type 3a (44%); type 3b (56%)	34 months	77.2	No failure	Complication incidence (18.8%), deep venous thrombosis (6.3%)
**Our current series**	18	8/10	68.3	Type 3a (61.1%); type 3b (38.9%)	61.0 months	73.7	No failure	Complication incidence (33.3%), dislocation (16.7%), delay wound healing (16.7%)

*mHHS* modified Harris Hip Score

For all patients, the mHHS significantly improved from an average preoperative value of 44.1 (rang 35 to 50) to 73.7 (rang 68 to 85) at the last follow-up, and mHHS was good in 2 (11.1%) hips, fair in 15 (83.3%) hips, and poor in 1 (5.6%) hip. The average preoperative UCLA score was 2.6 (rang 2 to 4), which improved significantly to 7.3 (range 7 to 8) at the last follow-up. The average SF-36 scores improved significantly at the last follow-up, especially in bodily pain category. The mean LLD decreased significantly from 24.2 mm (range 20 to 32 mm) preoperatively to 5.8 mm (range 3 to 9 mm) at the last follow-up. However, there was no significant difference between these two methods in terms of mHHS, UCLA, SF-36, LLD, and COR at the last follow-up (*p* > 0.05) (Table 3). No hips were outside the Lewinnek [20] acetabular cup abduction and anteversion safe range. We discovered bone graft incorporation in all hips one year after the revision operation. Asymptomatic grade-1 heterotopic ossifications were found in 3 (16.7%) patients who received no advanced treatment. No failure for acetabular loosening or metal failure was found at the last follow-up.

**Table 3:** Comparison of preoperative and the last follow-up results

Variables	Total patients	Double-TM cups alone	Technique combined with IBG	***p*** value
**mHHS**^**△**^	29.6 ± 5.09	28.5 ± 4.14	31.0 ± 5.90	0.128
**UCLA**^**△**^	4.7 ± 0.46	4.8 ± 0.42	4.6 ± 0.52	0.423
**SF-36**^**△**^				
Physical functioning	6.1 ± 1.86	5.7 ± 1.70	6.5 ± 2.07	0.341
Role-physical	19.7 ± 2.87	20.4 ± 2.99	18.8 ± 2.61	0.203
Bodily pain	35.4 ± 3.26	36.3 ± 3.74	34.3 ± 2.25	0.179
General health	7.8 ± 2.62	7.7 ± 3.09	8.0 ± 2.07	0.821
Vitality	11.6 ± 2.75	11.5 ± 3.03	11.8 ± 2.55	0.786
Social functioning	8.9 ± 2.90	9.5 ± 3.60	8.3 ± 1.67	0.141
Role-emotional	10.9 ± 3.93	11.5 ± 2.92	10.3 ± 5.06	0.263
Mental health	6.2 ± 2.96	6.7 ± 3.68	5.6 ± 1.77	0.788
**LLD** (mm)^**△**^	18.3 ± 2.59	18.4 ± 2.99	18.3 ± 2.19	0.928
**COR**^**a**^	1.08 ± 0.12	1.07 ± 0.13	1.08 ± 0.11	0.964
**COR**^**b**^	1.06 ± 0.11	1.05 ± 0.11	1.06 ± 0.12	0.858

^**△**^Improvement between the last follow-up and pre-operation

^**a**^Vertical position of COR/contralateral position of COR (the last follow-up)

^**b**^Horizontal position of COR/contralateral position of COR (the last follow-up)

*TM* trabecular metal, *IBG* impacting bone grafting, *mHHS* modified Harris Hip Score, *UCLA* University of California, Los Angeles activity score, *SF-36* 36-item Short Form Health Survey, *LLD* limb-length discrepancy, *COR* center of rotation

## Discussion

Acetabular revision that involves complex bone defects presents a complex and challenging procedure for the arthroplasty surgeon. Multiple surgical reconstruction options have been described for acetabular revision, including jumbo cup component, IBG combined with a cemented cup, metal mesh, bulk autograft, or allograft combined with hemispherical cups, cup-cage construct, and TM augments and hemispherical cups [2, 7]. Using jumbo cup is a straightforward method to reconstruct Paprosky III acetabular defect with a good survival rate of 96% at 15 years of follow-up [9]. However, the host bone would be widely removed when a jumbo cup was used, and it would be difficult to reconstruct with the residual bone stock at the second revision surgery. To fill the bone defects adequately and maximize the contact with the host bone, custom-made implants are one option to address large bone defects in revision surgery [27]. TM components could provide strong initial stability and promote a deep bony ingrowth with the design of high porosity and low modulus of elasticity [26]. Currently, some studies have been carried out to evaluate the effects of a novel treatment strategy of double-cup technique applied to manage Paprosky III acetabular defects without pelvic discontinuity, and double-TM cups reconstruction was considered a credible way for acetabular revision according to their short-term results [7, 12]. In a word, there are multiple options for acetabular revision recently, but with no clear optimal treatment described.

We used double-TM cups alone or combined with IBG to performed revision surgery and achieved good mid-term clinical and radiological results in patients with Paprosky III acetabular defects without pelvic discontinuity. Clinically, patients receiving double-TM cups showed a considerable improvement in a variety of functional scores. Double-TM construct was associated with an average of a 29.6-mHHS and a 4.7-UCLA increase between preoperative and the last follow-up. In this study, the average mHHS score of 73.7 points at the last follow-up was similar to the previous research results with double-cup technique [7, 12] (Table 2). Furthermore, the improvement in the mHHS of double-TM construct is similar to the improvement achieved with alternative treatment options. For example, porous tantalum shells and augments have a reported average postoperative mHHS of 81 [13]. Custom triflange acetabular component and uncemented jumbo cups have a reported average postoperative mHHS of 76.2 and 78.5, respectively [28].

Radiographically, restoring the hip COR to an anatomic position is important to re-create normal biomechanics and decrease joint reactive force and to improve the wear resistance and longevity of the acetabular construct [5, 6]. In our present study, we have used double-TM cups alone or combined with IBG to achieve a reduction of the migration of hip COR. Despite the large and complex of acetabular bone defects, we wished the postoperative ratio of position of COR in surgical site/contralateral site to be more close to 1. The results of our study showed the hip COR was corrected to be more similar with contralateral site and there was no significant difference between these two methods (Table 3). It is vitally important, in fact, that surgeons should pay more attentions to achieving initial stability of implants, biologic ingrowth, and normal anatomical structure in acetabular revision [4].

In revision surgery with large acetabular defects, the complications rate is also an important issue. The complication incidence (33.3%) of our present study was higher than that of what Loppini et al. reported (18.8%) [12] but was similar to that of what Webb et al. reported (40%) [7] (Table 2). Our complication rate, however, was similar to alternative treatment options. Custom triflange acetabular component have a reported complication rate of 29% [28]. The dislocation rate of revision THA ranged from 14 to 21% [29]. Among the reported complication of our study, dislocation was the most common (16.7%). The dislocation rate of our study was similar to the treatment option with augments for complex bone defects [30], but reporting was heterogeneous across studies. Revision surgery using cementless jumbo cups, reinforced cages and rings, and custom triflange acetabular component have a dislocation rate of 8.3%, 7.2%, and 11%, respectively [28, 31]. Furthermore, we found the dislocation rate for acetabular revision (33.3%) was higher than that for revision THA (8.3%). It has been demonstrated that patient-derived factors, surgical factors, or both influences the rate of THA dislocation [32]. We believe the previous surgery and posterolateral surgical approaches are risk factors for dislocation following revision THA. The procedure of enhanced posterior soft tissue repair could reduce the dislocation rate [32]. Similarly, the malposition of the acetabular cup and femoral head size are common surgical factors for dislocation in revision THA [32]. With the help of preoperative 3D simulation and model, complex revision THA could be managed with moderate to high accuracy and satisfied clinical outcomes [26]. Larger femoral heads could reduce the rate of dislocation, so all patients in our study were applied with 32-mm heads. Currently, several studies have reported that dual mobility bearing articulations have low rates of dislocation after revision THA [32, 33]. Therefore, detailed preoperative evaluation, reasonable surgical procedure and components, and postoperative assessment are the keys to reduce the dislocation after revision THA.

Due to the large and complex acetabular bone defects, a gap exists between the socket and the host bone during revision THA. Undoubtedly, poor quality of acetabular bone bed would cause reconstruction failure. Currently, IBG combined with acetabular components was widely used in revision THA when significant bone defects exist [17]. The technique of IBG would take the place of the bulk grafts gradually because of low osseointegration potential of bulk grafts [10]. One of the major benefits of IBG is the ability to restore bone stock. Garcia-Cimbrelo et al. [34] have reported that IBG contributed to restore acetabular bone stock and anatomic hip COR, and had good results in the last follow-up. However, autografts were difficult to widely use because of donor shortage and donor site morbidity. By the technique of creeping substitution, vascularization and incorporation occurred in impacting allografts [35]. But some studies showed poor outcomes in revision THA with structural bone grafts without reinforcement devices [11]. Therefore, many authors recommended the combined application of other revision devices to gain good to excellent clinic and radiographic outcomes [14]. In our present study, eight (44.4%) patients underwent reconstruction with double-TM cups combined with IBG, and we found bone graft incorporation in all hips 1 year after the revision surgery. In terms of the clinical and radiological assessments after revision surgery, there was no significant difference between the method of double-TM cups alone and the method of double-TM cups combined with IBG at the last follow-up. Therefore, this reconstruction method of double-TM cups combined with IBG frequently allows the surgeons to restore the anatomic hip COR and improve the biomechanics of the hip in revision surgery.

Furthermore, the cost of revision THA is usually an important issue, especially in patients of large and complex bony defects. Usage of TM augments, cup-cage, or custom triflange implant brings great financial burden to patients, particularly in developing countries. Although the charge standard and medical reimbursement policy of different areas and countries are different, it is comparable in one institution of a country. In our institution, the cost of double-TM cups with/without bone graft was less than the use of one TM cup with augments or with the cup-cages components, or with custom triflange construct. Therefore, we have reconstructed Paprosky III acetabular defects with double-TM cups alone or in combination with IBG, and the results revealed good to excellent mid-term clinical function with less cost finally in our present study.

We acknowledge limitations of our study. First, our present study was retrospectively designed with relatively small sample size. It was hard to obtain a larger number of patients who underwent rare and uncommon acetabular reconstruction with double-TM cups alone or combined with IBG from a single institution. Second, the mixed pathology of patients could contribute to inconsistent results. Likewise, the implants of femur revision in this series were not identical. We chose implants according to the anatomy and requisite during the operation. Finally, the relatively short to mid-term follow-up period also could contribute to inferior results. Further study with longer follow-up are needed to determine the long-term results of the use of double-TM cups with/without IBG for complex acetabular reconstruction.

## Conclusion

In summary, acetabular revision surgery with double-TM cups alone or combined with IBG can achieve similar outcomes in terms of their clinical and radiological results. Therefore, there is no doubt that these two methods are practical and reliable techniques to reconstruct complex acetabular bone defects. Nevertheless, the overall complications rate was still a challenging technique. To avoid high complications, detailed preoperative design and nearly anatomic construction should be performed.

## Data Availability

All data generated or analyzed during this study are included in this published article.

## Abbreviations

TM: Trabecular metal
double-TM: Double trabecular metal
IBG: Impaction bone grafting
THA: Total hip arthroplasty
COR: Center of rotation
MSIS: Musculoskeletal Infection Society
mHHS: Modified Harris Hip Score
SF-36: Short Form 36
UCLA: University of California, Los Angeles
LLD: Limb-length discrepancy
BMI: Body mass index
PJI: Periprosthetic joint infection
IQR: Interquartile ranges
